# Constitutive Overexpression of Muscarinic Receptors Leads to Vagal Hyperreactivity

**DOI:** 10.1371/journal.pone.0015618

**Published:** 2010-12-22

**Authors:** Angelo Livolsi, Nathalie Niederhoffer, Nassim Dali-Youcef, Walid Mokni, Catherine Olexa-Zorn, Jean-Pierre Gies, Luc Marcellin, Josiane Feldman, Pascal Bousquet

**Affiliations:** 1 Laboratoire de Neurobiologie et Pharmacologie Cardiovasculaire (EA 4438), Université de Strasbourg, Strasbourg, France; 2 Pôle Pédiatrique Médico-Chirurgical, Hôpitaux Universitaires de Strasbourg, Strasbourg, France; 3 Laboratoire de Biophotonique et de Pharmacologie, UMR CNRS 7213, Université de Strasbourg, Strasbourg, France; 4 Institut de Génétique et de Biologie Moléculaire et Cellulaire de Strasbourg (IGBMC), INSERM/CNRS/Université de Strasbourg, Illkirch, France; 5 Laboratoire de Biochimie Générale et Spécialisée, Hôpitaux Universitaires, Strasbourg, France; 6 Centre d'Investigation Clinique, Hôpitaux Universitaires-INSERM, Strasbourg, France; 7 Département d'Anatomie Pathologique, Hôpitaux Universitaires, Strasbourg, France; Alcon Research, Ltd, United States of America

## Abstract

**Background:**

Alterations in muscarinic receptor expression and acetylcholinesterase (AchE) activity have been observed in tissues from Sudden Infant Death Syndrome (SIDS). Vagal overactivity has been proposed as a possible cause of SIDS as well as of vasovagal syncopes. The aim of the present study was to seek whether muscarinic receptor overexpression may be the underlying mechanism of vagal hyperreactivity. Rabbits with marked vagal pauses following injection of phenylephrine were selected and crossed to obtain a vagal hyperreactive strain. The density of cardiac muscarinic receptors and acetylcholinesterase (AchE) gene expression were assessed. Blood markers of the observed cardiac abnormalities were also sought.

**Methodology/Principal Findings:**

Cardiac muscarinic M_2_ and M_3_ receptors were overexpressed in hyperreactive rabbits compared to control animals (2.3-fold and 2.5-fold, respectively) and the severity of the phenylephrine-induced bradycardia was correlated with their densities. A similar overexpression of M_2_ receptors was observed in peripheral mononuclear white blood cells, suggesting that cardiac M_2_ receptor expression can be inferred with high confidence from measurements in blood cells. Sequencing of the coding fragment of the M_2_ receptor gene revealed a single nucleotide mutation in 83% of hyperreactive animals, possibly contributing for the transcript overexpression. Significant increases in AchE expression and activity were also assessed (AchE mRNA amplification ratio of 3.6 versus normal rabbits). This phenomenon might represent a compensatory consequence of muscarinic receptors overexpression. Alterations in M_2_ receptor and AchE expression occurred between the 5th and the 7th week of age, a critical period also characterized by a higher mortality rate of hyperreactive rabbits (52% in H rabbits *versus* 13% in normal rabbits) and preceeded the appearance of functional disorders.

**Conclusions/Significance:**

The results suggest that cardiac muscarinic receptor overexpression plays a critical role in the development of vagal hyperreactivity, whereas AchE hyperactivity appears as a compensatory consequence of it. Since similar vagal disorders were observed recently by us in SIDS, muscarinic receptor overexpression could become a marker of risk of vasovagal syncopes and SIDS.

## Introduction

We have recently reported alterations in muscarinic receptor expression and acetylcholinesterase (AchE) activity in tissues from Sudden Infant Death Syndrome (SIDS) [1], a syndrome for which vagal overactivity has been proposed as a potential risk factor [2]. Vagal overactivity has been proposed as a possible cause of vasovagal syncopes [3], [4], [5], which remain a challenge in many patients, in particular infants and young athletes, and account for 1-2% of all emergency department visits [6], [7]. Vasovagal syncope has a lifetime cumulative influence of 35% and the frequency of syncope in students ranges from 20 to 50% [8], [9]; it may be involved in sudden death, not only in newborns as stated above, but also in young athletes [10], [11].

Therefore, vagal overactivity appears as a possible common cause of various pathological processes. Muscarinic receptor overexpression, as we described in SIDS, could be a key factor for the development of vagal overactivity.

We previously described an animal model of vagal hyperreactivity [12]. In the present study, we used this rabbit model to explore the pathogenesis of vagal hyperreactivity. As in SIDS, the density of muscarinic receptors as well as AchE expression were increased in the hearts of hyperreactive rabbit. Seeking blood markers, we found that lymphocytes and heart exhibited the same cholinergic abnormalities. In addition, we describe the evolution over age of the biochemical features observed in lymphocytes.

## Materials and Methods

### Animals and *in vivo* experiments

The experimental rabbit model of vagal hyperreactivity has been described previously [12]. Data obtained in hyperreactive (H) animals (12–14 weeks old) were compared to those obtained in age-matched normal (N) rabbits.

Maximal R-R interval was used to assess the baroreflex function in conscious animals. We therefore measured the duration of the R-R interval on ECG recording after injection of given dose of phenylephrine (PNE) (500 µg kg^−1^) into the marginal ear vein as described previously [12]. Maximal R-R interval appeared 2 to 3 beats after the end of the ramp of blood pressure. In all experiments, animals were pretreated with the beta-adrenergic receptor antagonist, propranolol (100 µg kg^−1^) in order to avoid sympathetic arrhythmogenic influences on heart responses. In some experiments, the AchE inhibitor neostigmine (25 µg kg^−1^) was also administered prior to PNE; in these experiments, PNE doses were reduced to 250 µg kg^−1^. All drugs were injected intravenously.

At the end of *in vivo* experiments, rabbits were sacrificed with a bolus injection of pentobarbital (50 mg kg^−1^) and the hearts were removed. Tissue samples were stored at −20°C in 0.25 M saccharose for radioligand binding experiments or immediately frozen in liquid nitrogen and then stored at −80°C for AchE gene expression measurements.

### Radioligand binding experiments

Methods were adapted from Gies et al. [13], [14]. All saturation binding experiments were carried out at 25°C in 0.5 mL Tris buffer (50 mM, pH 7.4) containing 50–70 µg protein. Heart samples were incubated for 90 min. Incubation was stopped by rapid vacuum filtration over Whatman GF/C glass microfibre filters (VWR International SAS, Strasbourg, France) presoaked with polyethylenimine 0.3% in order to reduce non-specific binding to the filters. Filters were washed twice with 4 mL of ice-cold incubation buffer and transferred to 6 mL counting vials containing 3 mL of scintillation cocktail (Ready Protein+™, Beckman Coulter France SA, Roissy CDG, France). Radioactivity was then counted in a liquid scintillation spectrometer (LS 6000SC; Beckman Instruments) with an efficiency of 45%. Scatchard analysis of the saturation data (linear regression with Excel Software) was used to yield the maximal specific binding sites (B_max_; fmol mg^−1^ protein). Protein content was measured according to the method of Bradford [15] using bovine γ-globulin as standard.

The density of total muscarinic receptors was assessed using the tritiated non-selective muscarinic receptor antagonist N-methylscopolamine ([^3^H]NMS; 81.0 Ci mmol^−1^) added in 12 concentrations ranging from 40 to 2000 pmol L^−1^. Non-specific binding was determined in the presence of 1 µmol L^−1^ atropine. M_1_, M_2_ and M_3_ muscarinic receptors were selectively labelled using tritiated pirenzepine ([^3^H]PZ; 86.0 Ci mmol^−1^), AF-DX 384 ([^3^H]AF-DX 384; 120.0 Ci mmol^−1^) and 4-diphenylacetoxy-*N*-methyl piperidine methiodide ([^3^H]4-DAMP; 80.1 Ci mmol^−1^), respectively [16], [17], all added in 12 concentrations ranging from 100 to 5000 pmol L^−1^. Nonspecific binding was determined in the presence of 10 µmol L^−1^ atropine.

### Preparation of peripheral mononuclear white blood cells (PMBC)

Three ml of fresh blood samples from normal and vagal hyperreactive rabbits were placed on 3 ml of Histopaque®-1077 (Sigma-Aldrich, Saint-Louis, MO) and centrifuged at 1500 rpm for 30 min. The white mononuclear cell layer was taken and transferred to tubes with 10 ml of 0.1 M sodium phosphate buffer pH 7.4 (PBS) and centrifuged at 1800 rpm for 10 min. Mononuclear cells were resuspended in 100 µl PBS and processed for total RNA extraction.

### M_2_ receptor and AchE gene expression

Total RNA was extracted from PMBC samples using the MagNA Pure Compact RNA Isolation Kit on a MagNA Pure Compact Instrument (Roche, Basel, Switzerland) following the manufacturer protocol instructions. One hundred and fifty ng of total RNA were then reverse transcribed into cDNA using the LightCycler® Transcriptor First Strand cDNA Synthesis Kit (Roche, Basel, Switzerland). Cardiac samples were homogenized in Qiazol® and RNA was extracted using Qiagen® (QIAGEN Inc., Valencia, CA) extract columns following the manufacturer's protocol instructions; 2 µg of total RNA were then reverse transcribed into cDNA using the superscript reverse transcriptase II (SSRII, Invitrogen Corp., France).

M_2_ and AchE gene expressions were measured by quantitative real time polymerase chain reaction (Q-RT-PCR) using a LightCycler® amplifier and a fluorescent SybrGreen I dye for detection (Roche, Basel, Switzerland) using specific primers for M_2_ receptors (forward 5′GGCAGGAATGATGATTGCAGC3′; reverse 5′AGCTAGTTGGGTCTTCAGGTC3′), AchE (forward 5′CCCAAGAAAGCATCTTCCGCT3′; reverse 5′TGAGGGTACCTATTTTCTGG3′), and the rabbit 18S housekeeping gene (forward 5′CGCGGTTCTATTTTGTTGGT3′; reverse 5′CGAAAGTCGGAGGTTTGAAG 3′), used for normalization. The cycling conditions were: 95°C for 10 min, then 45 cycles of 95°C for 15 sec, 60°C for 10 sec and 72°C for 20 s. Specificity of amplification products was assessed by melting curve analysis. Relative M_2_ and AchE gene expressions were quantified using the 2^−ΔΔ*C*t^ method [18].

### M_2_ receptor gene sequencing

Genomic DNAs from normal and vagal hyperreactive rabbits were isolated from peripheral blood cells using Qiagen technology (GmbH, Hilden, Germany) and quantified by spectrophotometry. Exon of the cholinergic muscarinic M_2_ receptor gene (rabbit CHRM2, Ensembl genome database) was amplified using specific primers (forward 5′GGCAGGAATGATGATTGCAGC3′ and reverse 5′AGCTAGTTGGGTCTTCAGGTC3′). All amplification reactions were performed on a Mastercycler epgradient 5 (Eppendorf, France) using a Taq DNA polymerase from Sigma (Sigma-Aldrich, Saint-Quentin, France), 1.5 mM MgCl_2_ and 500 nM of each primer in a PCR buffer (Sigma-Aldrich, Saint-Quentin, France) under the following cycling conditions: 94°C for 5 min, followed by 35 cycles of 30 s at 94°C, 20 s at 58°C, 15 s at 72°C. Quality of the amplification products was checked by gel electrophoresis. After amplification, PCR products were purified on QIAquick columns (Qiagen) and processed on an AB 3100 genetic analyzer (Applied Biosystems, CA). After the sequencing step, extension products were size-fractionated by capillary electrophoresis and sequences were compared to the M_2_ receptor gene reference sequence from the Ensembl genome database using SeqScape (Applied Biosystems, CA) and BioEdit (Ibis Therapeutics, CA) softwares.

### AchE enzyme activity

AchE enzyme activity was measured in erythrocytes from venous total blood samples collected on heparin according to an enzymatic colorimetric assay as previously described [19].

### Ethics statement

All the methods employed in this work are in accordance with the French law concerning experimentations on vertebrate laboratory animals (*Décret 2001-464* from May 29, 2001 as a revision of the *Décret 87-848, 1987*) and according to European guidelines. PB, JF and JPG hold personal agreements from the Direction des Services Vétérinaires du Bas-Rhin, Agriculture Ministery, France (authorization numbers 67–249 to PB, 67-2010 to JF and 67–87 to JPG) which cover the protocols followed in the present study.

### Statistics

All values are expressed as mean ± standard deviation (SD). SD values were preferred to SEM since they better reflect variabilities of the measured parameters than SEM values. Unpaired *t*-tests were performed using the Mann-Whitney U test. *P* values < 0.05 were considered to be statistically significant.

## Results

### Haemodynamic effects of the standard dose of phenylephrine

The severity of vagal pauses was evaluated in conscious animals by measuring the duration of the R-R interval on ECG recording after intravenous injection of a standard dose of phenylephrine (PNE; 500 µg kg^−1^). The 500 µg kg^−1^ dose of PNE increased mean blood pressure by 44±11% (124±13 mmHg after PNE vs 81±3 mmHg at rest). Mean values of R-R interval duration after PNE challenge were 2339±786 ms and 10707±3421 ms in normal (N) and hyperreactive (H) rabbits, respectively.

### Expression of cardiac muscarinic receptors and correlation with the bradycardic response to phenylephrine

In saturation binding experiments, a significant 2.5- to 3-fold increase in the total density of muscarinic receptors (B_max_) labelled by [^3^H]NMS was observed in the heart of H rabbits as compared to controls (**Table 1**). To further characterize the muscarinic receptor sub-populations, M_1_, M_2_ and M_3_ receptors were selectively labelled using [^3^H]PZ, [^3^H]AF-DX 384 and [^3^H]4-DAMP, respectively. In the heart from rabbits with normal vagal responses, M_2_ receptors represented 40 to 45% of the total, and the remainder were M_3_; no expression of receptors of the M_1_-subtype could be detected (*n* = 3, data not shown). The densities of both M_2_ and M_3_ receptors were significantly and significantly enhanced in H rabbits as compared to N rabbits (2.3-fold and 2.5-fold, respectively, **Table 1**).

**Table 1:** Cardiac muscarinic receptors density and R-R interval duration in normal (N) and vagal hyperreactive (H) rabbits.

	N rabbits		H rabbits	
	B_max_[fmol mg^−1^ protein]	R-R interval[ms]	B_max_[fmol mg^−1^ protein]	R-R interval[ms]
**Total M**	157.6 (37.8)	1867 (615)	469.0 (185.5)	10644 (2633)
(*n* = 6 N and 9 H)			(*P* = 0.001)	(*P*<0.0001)
**M_2_**	65.6 (18.9)	2600 (783)	148.7 (72.6)	11560 (4339)
(*n* = 7 N and 10 H)			(*P* = 0.01)	(*P*<0.0001)
**M_3_**	88.6 (30.1)	2317 (939)	226.9 (77.3)	9960 (2954)
(*n* = 6 N and 10 H)			(*P* = 0.001)	(*P*<0.0001)

The severity of vagal pauses was evaluated in conscious animals by measuring the duration of R-R interval on the ECG recording after challenge with PNE 500 µg kg^−1^ following the procedure described in Material and Methods. Total, M_2_ and M_3_ muscarinic receptors densities (B_max_) in hearts from normal (N) and vagal hyperreactive (H) rabbits were obtained from Scatchard analysis of the saturation data using [^3^H]NMS (total), [^3^H]AF-DX 384 (M_2_) and [^3^H]4-DAMP (M_3_) as radioligands. Binding conditions were as described in Material and Methods. Values are means (SD) of n independent experiments. P: H versus N within each group.

**Figure 1:**
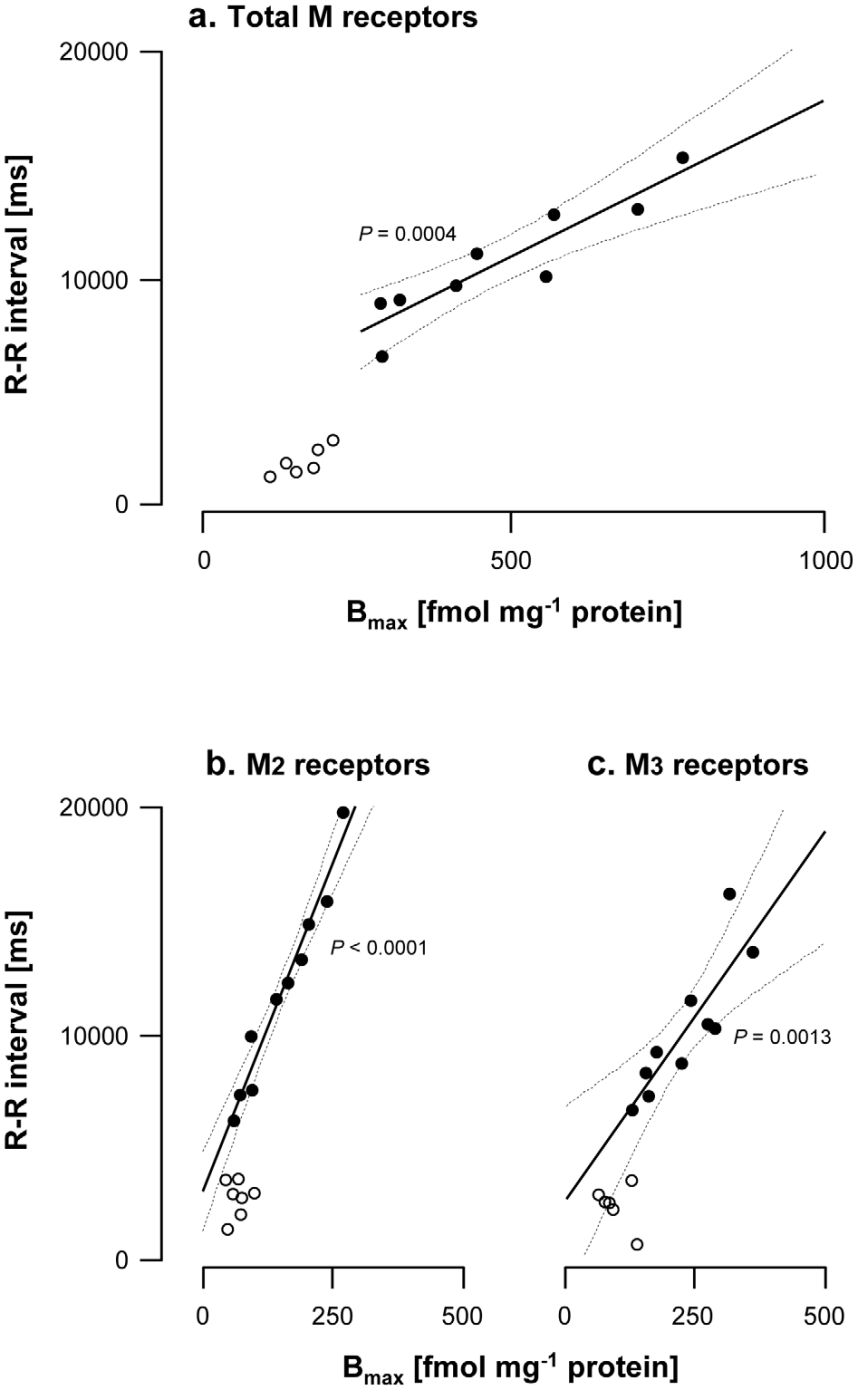
Correlations between R-R interval and total, M_2_ and M_3_ muscarinic receptor density in hearts from normal (N) and vagal hyperreactive (H) rabbits. R-R intervals were measured in conscious rabbits challenged with PNE 500 µg kg^−1^ following the procedure described in Material and Methods. Total, M_2_ and M_3_ muscarinic receptor densities in hearts (B_max_; fmol mg^−1^ protein) were determined from Scatchard analysis of the saturation data using [^3^H]NMS, [^3^H]AF-DX 384 and [^3^H]4-DAMP, respectively, as radioligands. Binding conditions were as described in Material and Methods. Each symbol represents one animal. (**a**) Total muscarinic receptors; *n* = 9 H (full symbols) and 6 N (open symbols). (**b**) M_2_ muscarinic receptors; *n* = 10 H (full symbols) and 7 N (open symbols). (**c**) M_3_ muscarinic receptors; *n* = 10 H (full symbols) and 6 N (open symbols).

The R-R interval duration was taken as a measure of the bradycardic response to PNE. In H rabbits used for [^3^H]NMS binding experiments, the R-R interval durations, after injection of PNE, ranged from 6500 to 15200 ms (mean 10644±2633) compared to N rabbits (1200 to 2800 ms; mean 1867±615). The 2.5- to 3-fold increase in the density of muscarinic receptors was accompanied by a 4.5 to 5-fold increase in R-R duration (**Table 1**). Within this group of animals, a highly significant positive correlation was found between the R-R interval and the total muscarinic receptor density (**Fig. 1a**); compared to the control group, all values for receptor density (and consequently for R-R interval) were shifted to higher levels.

Similarly, the R-R interval was significantly correlated with the density of M_2_ and M_3_ receptors within the two respective groups (range of R-R interval values from 5800 to 19600 ms, mean 11560±4339, and from 6400 to 16000 ms, mean 9960±2954, for animals used for M_2_ and M_3_ binding experiments, respectively) (**Fig. 1b,c**). The slopes of the linear regressions differed markedly, however, being almost 2-fold greater with M_2_ than M_3_, indicating that a change in M_2_ receptor density influenced cardiac responses much more than the same change in M_3_ receptor density. Thus, it appears that vagal hyperreactivity can be considered a consequence of the overexpression of cardiac M_2_ and M_3_ muscarinic receptors.

### Expression of muscarinic receptors in peripheral mononuclear white blood cells

**Figure 2:**
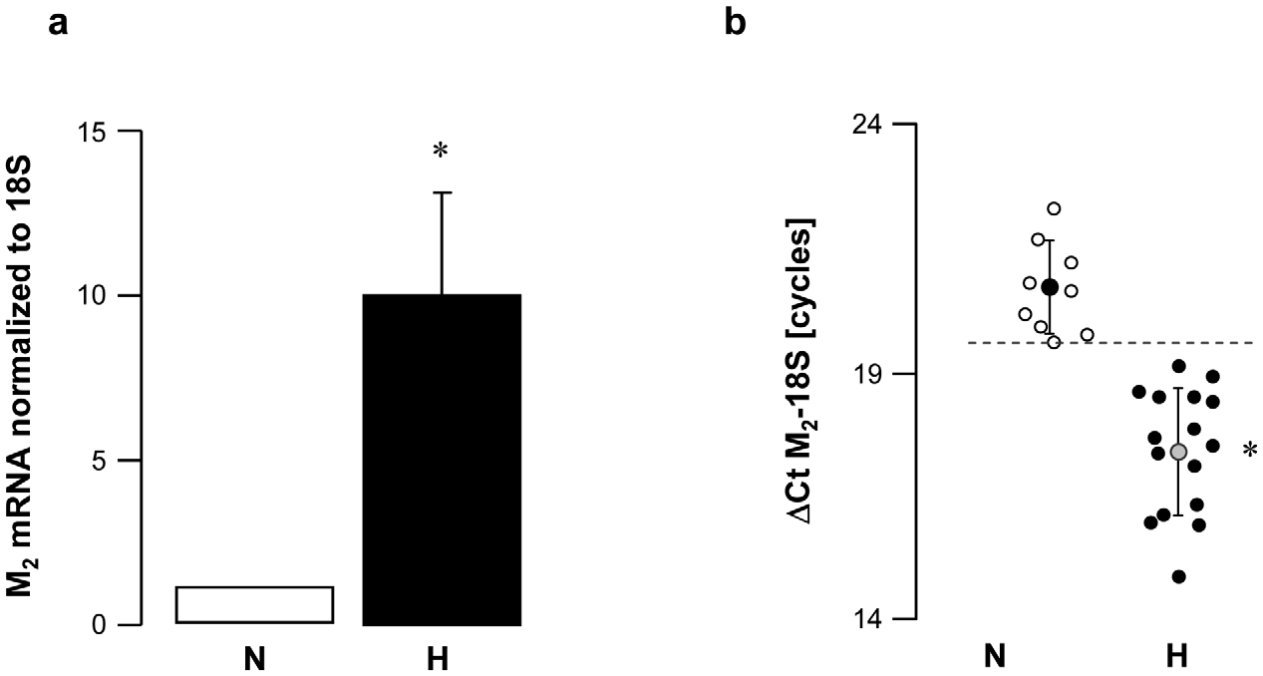
M_2_ muscarinic receptor gene expression in peripheral mononuclear white blood cells from normal (N) and vagal hyperreactive (H) rabbits. R-R intervals were measured in conscious rabbits challenged with PNE 500 µg kg^−1^ following the procedure described in Material and Methods. M**_2_** muscarinic gene expression was assessed in peripheral mononuclear white blood cells by quantitative RT-PCR and normalized to the rabbit 18S housekeeping gene. Values in (**a**) show amplification ratio calculated according to the 2^−ΔΔ*C*t^ method of 9 (N) and 16 (H) experiments. In (**b**), each symbol represents one animal; ΔCt M**_2_**-18S corresponds to the number of amplification cycles needed to detect M**_2_** fluorescence standardized to 18S; thus, the lower the ΔCt M_2_-18S, the greater M**_2_** mRNA initial quantity. *: *P*<0.0001 *versus* N.

M_2_ mRNA expression level was about 10 times higher in vagal hyperreactive rabbits compared to that observed in normal rabbits (**Fig. 2a**). Compared to normal animals, M_2_ mRNA relative quantities were increased in all rabbits displaying baroreflex dysfunction and vagal hyperreactivity (**Fig. 2b**).

### AchE gene expression and enzyme activity

In an attempt to better characterise vagal disorders in H rabbits, we looked at whether AchE gene expression and enzyme activity were also changed in these animals.

**Figure 3:**
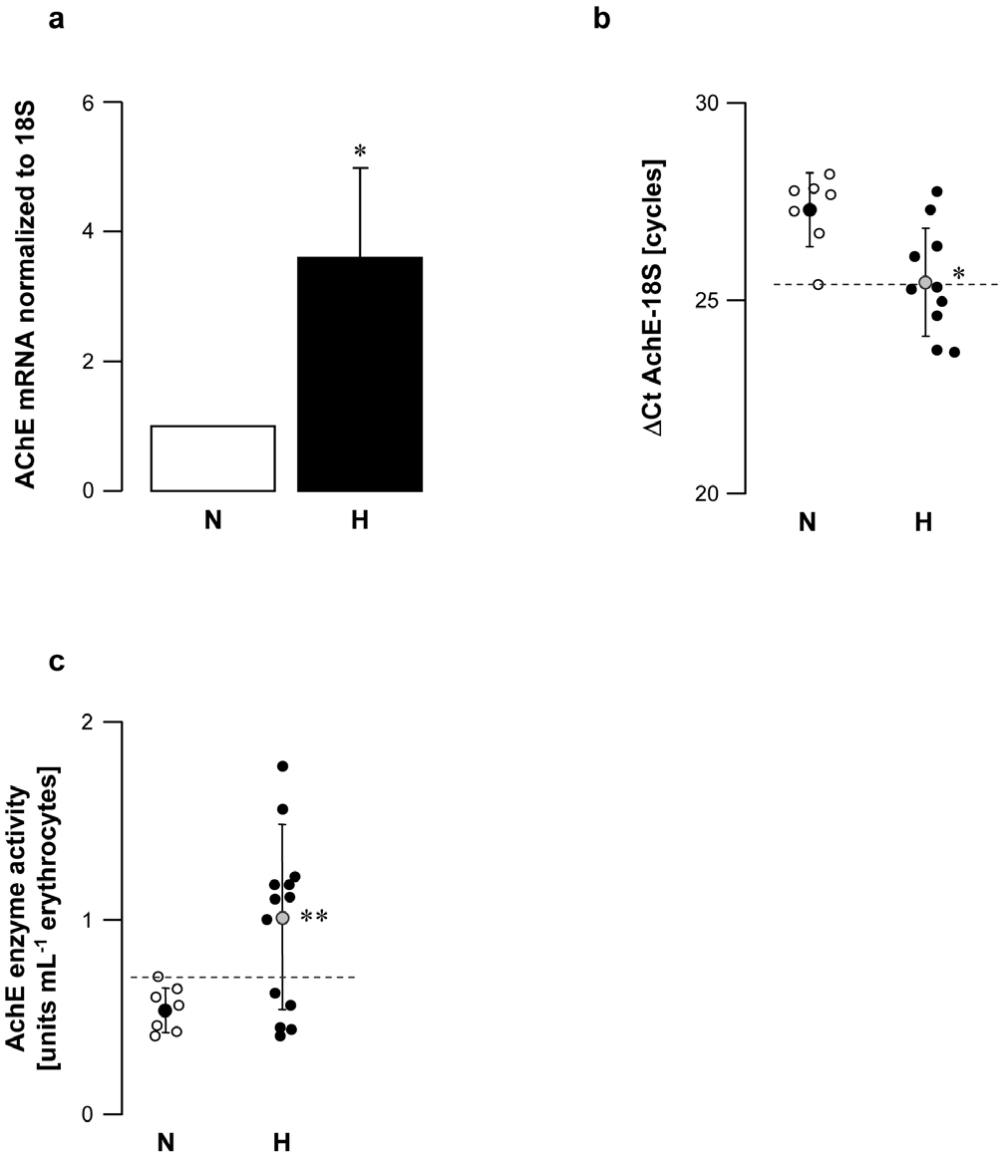
AchE cardiac gene expression and enzyme activity in erythrocytes from normal (N) and vagal hyperreactive (H) rabbits. R-R intervals were measured in conscious rabbits challenged with PNE 500 µg kg^−1^ following the procedure described in Material and Methods. (**a,b**) Cardiac AchE gene expression was assessed by quantitative RT-PCR and normalized to the rabbit 18S housekeeping gene. Values in (**a**) show amplification ratio calculated according to the 2^−ΔΔ*C*t^ method of 7 (N) and 10 (H) experiments. In (**b**), each symbol represents one animal; ΔCt AchE-18S corresponds to the number of amplification cycles needed to detect AchE fluorescence standardized to 18S; thus, the lower the ΔCt AchE-18S, the greater AchE mRNA initial quantity. (**c**) AchE enzyme activity was assayed colorimetrically in erythrocyte hemolysates from 7 (N) and 13 (H) animals. *: *P* = 0.008 *versus* N. **: *P* = 0.02 *versus* N.

**Figure 4:**
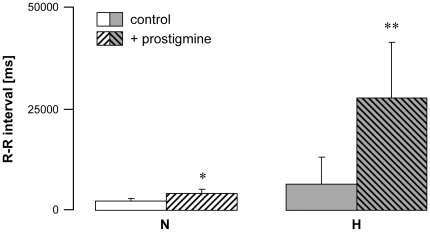
Effect of AchE enzyme blockade in normal (N) and vagal hyperreactive (H) rabbits. R-R intervals were measured in conscious rabbits challenged with PNE 250 µg kg^-1^ before (control) and after administration of neostigmine 25 µg kg^−1^. Values are means ± SD of 4 N and 4 H experiments. *: *P* = 0.02 *versus* N control; **: *P* = 0.03 *versus* H control.

Vagal hyperreactive rabbits displayed an AchE mRNA amplification ratio of 3.6 versus normal rabbits (**Fig. 3a,b**); this was associated with twice the enzyme activity in erythrocytes (**Fig. 3c**). When AchE was blocked by intravenous administration of neostigmine, a specific AchE inhibitor, H rabbits displayed massively increased bradycardia following PNE injection (up to 10-fold higher in some individuals; **Fig. 4**). These data confirmed that AchE enzyme activity was increased in the hearts of these animals. Taken together, the findings showed up-regulation of AchE in H rabbits.

### Evolution of vagal disorders with age

**Figure 5:**
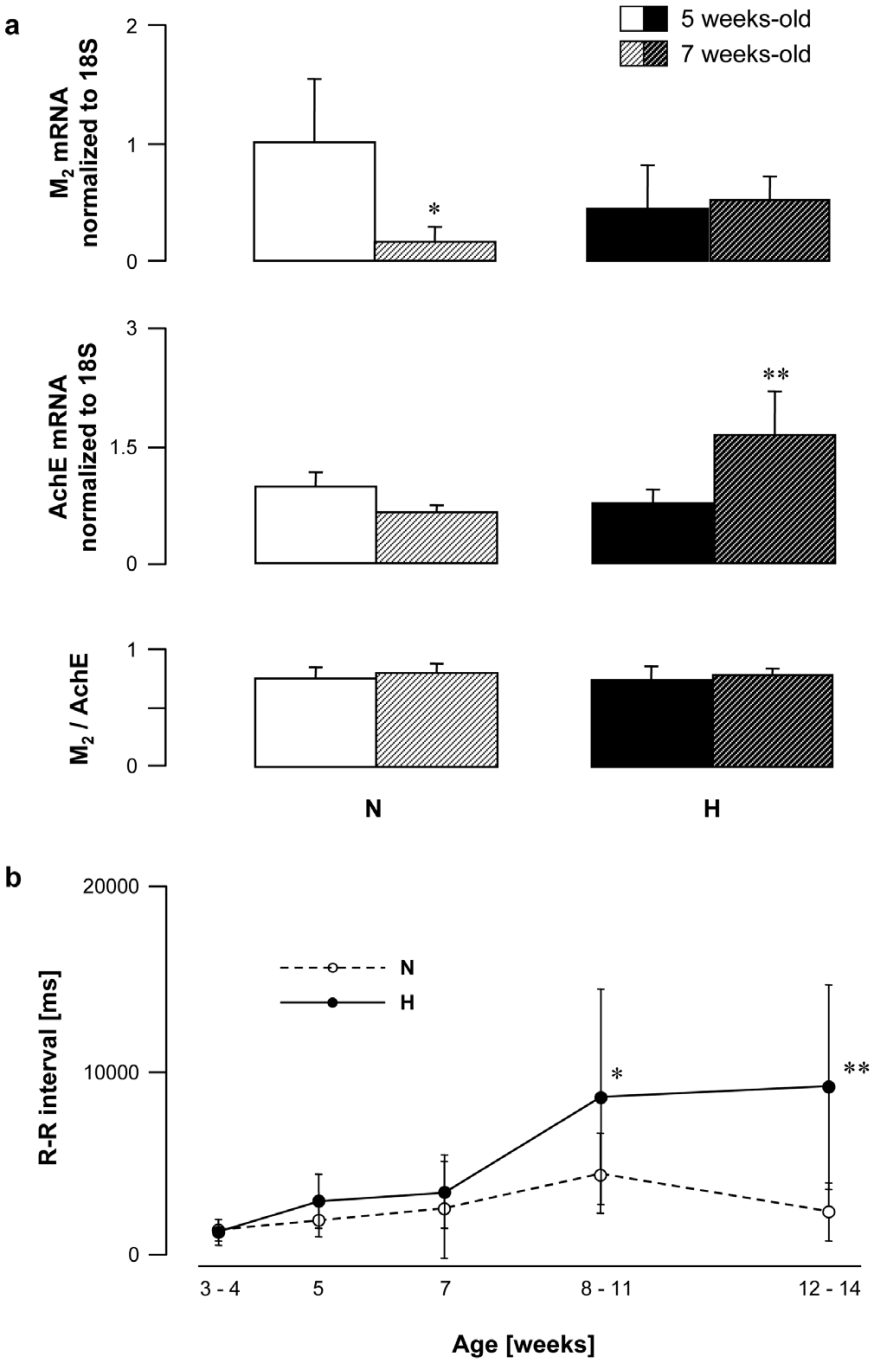
Age-dependent changes in R-R interval and M_2_ muscarinic receptor and AchE gene expression in peripheral mononuclear white blood cells from normal (N) and vagal hyperreactive (H) rabbits. (**a**) M_2_ muscarinic receptor and AchE gene expression were assessed in peripheral mononuclear white blood cells by quantitative RT-PCR and normalized to the rabbit 18S housekeeping gene. Values show amplification ratio calculated according to the 2^−ΔΔ*C*t^ method of 8–9 (N) and 7–11 (H) experiments. *: *P* = 0.0262 and **: *P* = 0.0122 *versus* 5 weeks-old rabbits within the N and the H group, respectively. (**b**) R-R intervals were measured in conscious rabbits challenged with PNE 500 µg kg^−1^ following the procedure described in Material and Methods. Values are means ± SD of 11 N and 15 H rabbits. *: *P* = 0.0179 and **: *P* = 0.0110 *versus* 7 weeks-old rabbits within the H group.

As a next step, age-dependent changes in vagal disorders and their functional consequences on R-R intervals were assessed in normal and hyperreactive rabbits. Blood samples were collected at the ages of 5 and 7 weeks. The M_2_ expression decreased between the 5th and the 7th week of age in rabbits with normal vagal responses, whereas it remained unchanged in hyperreactive animals (**Fig. 5a**). In contrast, the AchE expression remained stable in N rabbits but increased significantly in H rabbits within the same period (**Fig. 5a**). Consequently, the M_2_/AchE ratio was similar whatever the age and the strain (**Fig. 5a**).

Functionnal disorders appeared after 7 weeks of age: R-R interval duration was similar in all rabbits up to 7 weeks and then increased in H rabbits, while remaining stable in N animals (**Fig. 5b**). Despite SD error-bars appear overlapping on this figure, data were significantly different (P = 0.0179 at 8–11 weeks and P = 0.011 at 12–14 weeks compared to values observed at 7 weeks).

### Mortality study

Mortality has been assessed in a population of 2150 normoreactive rabbits and 385 hyperreactive animals.

In hyperreactive animals, the altered changes in M_2_ muscarinic receptor and AchE expression were associated with an abnormally high mortality rate between 5 to 7 weeks of age: 52% in H rabbits *versus* 13% in normal rabbits. Mortality was higher in male than female (57% *versus* 43%). In most cases, diarrhea and digestive dysfunctions were observed shortly before death.

### M_2_ receptor gene sequencing

**Figure 6:**
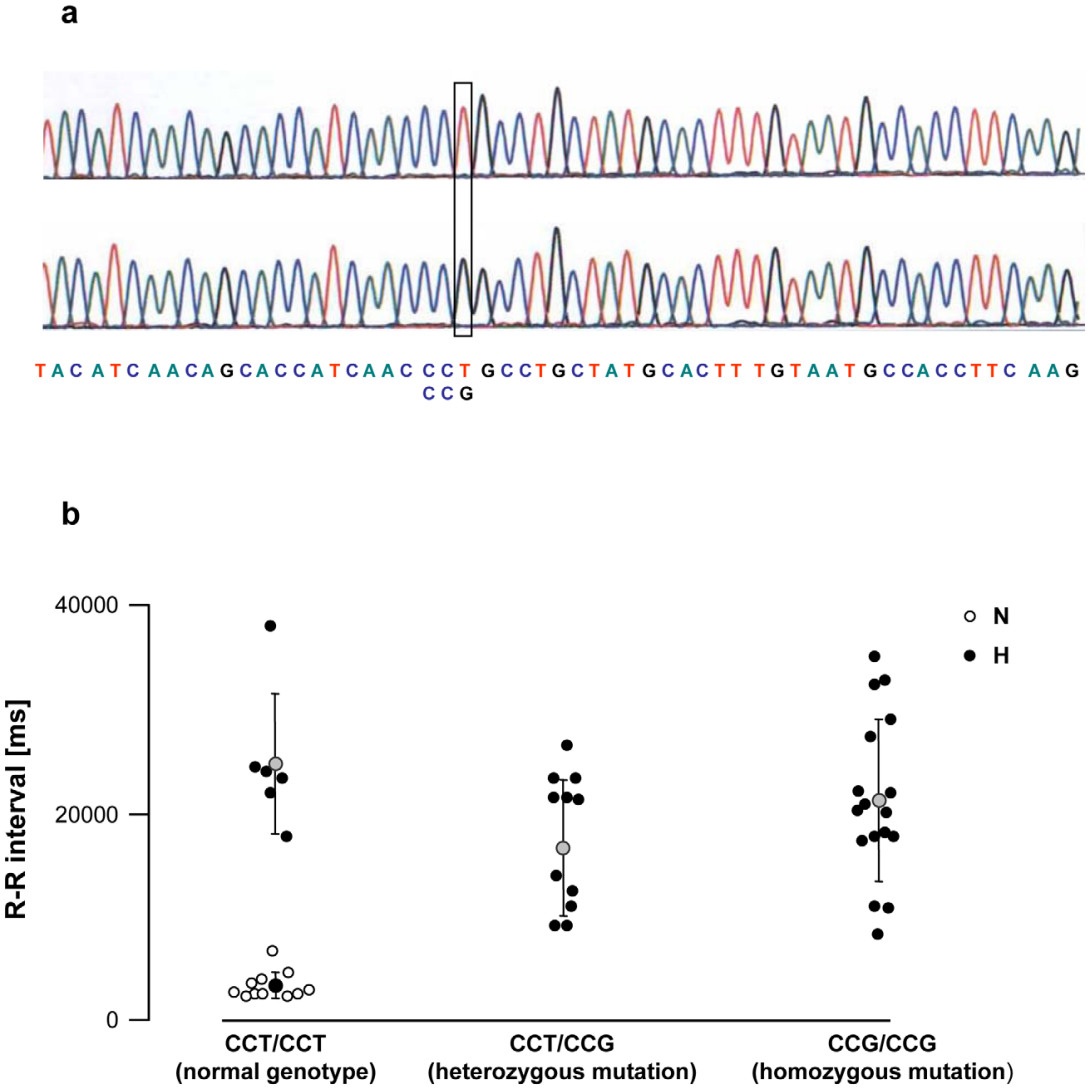
Polymorphism of the M_2_ cholinergic muscarinic receptor gene of normal (N) and vagal hyperreactive (H) rabbits. (**a**) DNA sequence analysis of the coding fragment of the M_2_ muscarinic receptor gene (Chmr2) showing the normal CCT codon and the single nucleotide substitution at position 1311 (G instead of T). (**b**) The severity of vagal pauses was evaluated in conscious animals by measuring the duration of R-R interval on the ECG recording after challenge with PNE 500 µg kg^−1^ following the procedure described in Material and Methods. DNA sequence analysis of the coding fragment of the M_2_ muscarinic receptor gene was carried out as described in Material and Methods. Each symbol represents one animal.

After sequencing the cholinergic receptor M_2_ gene, we detected a single nucleotide mutation from thymine to guanine (T→G) in position 1311 (**Fig. 6a**). This mutation transformed the normal CCT codon into CCG, both encoding for the proline amino-acid. The two codons were not equally distributed among animals. Over the 46 rabbits tested, 17 had the wild genotype CCT/CCT, 12 were heterozygous CCT/CCG and 17 were homozygous CCG/CCG. None of the 11 normal rabbits had the mutation, while it was present in 29/35, *i.e.*, 83% of the vagal hyperreactive rabbits (**Fig. 6b**). The mean R-R interval in normal rabbits was 3318±1336 ms; mean R-R intervals in hyperreactive rabbits were 24700±6855 ms (wild genotype CCT/CCT), 16650±6572 ms (heterozygous CCT/CCG) and 21096±7759 ms (homozygous CCG/CCG) (**Fig. 6b**).

## Discussion

In a previous study [12], we showed that spontaneous vagal pauses were observed in a particular strain of adult rabbits (12–14 weeks of age) which can therefore be used as an experimental model of vagal hyperreactivity. In the experimental conditions of the present study, the PNE test was used to screen hyperreactive animals. To prevent sudden death due to arrhythmogenic complications of PNE, the animals were treated with the β-blocking drug, propranolol. A unique bolus dose of the latter was delivered shortly before the PNE test. As shown previously, maximal R-R interval is a reliable index to assess the vagal reactivity in these animals [12].

In binding experiments, we showed that the densities of both M_2_ and M_3_ muscarinic receptors were enhanced in the heart of rabbits displaying exacerbated vagal responses. A similar increase of M_2_ mRNA was observed in peripheral mononuclear white blood cells. As the sequence of the M_3_ receptor gene was not known, PCR experiments regarding mRNA expression could not be performed. A significant correlation was established between the severity of the bradycardia and the cardiac muscarinic receptor expression level indicating that vagal hyperreactivity was highly dependent on muscarinic receptor density. The increase in the M_2_ subtype is in line with the well-established negative chronotropic effects of these receptors. We also found a significant increase in M_3_ receptor expression. M_3_ receptors have been identified in hearts of several mammalian species, including humans [16], [20]–[22], and recent studies showed that M_3_ stimulation mediates K^+^ currents in cardiac cells [23], [24]. Interestingly, no overlap between the muscarinic receptor expression in normal and diseased animals was observed and muscarinic receptor overexpression was detected in all hyperreactive animals, suggesting that it is a primary cardiac abnormality underlying vagal hyperreactivity.

Compared to control animals, bradycardia after PNE challenge was unexpectedly accentuated upon AchE blockade by neostigmine in hyperreactive rabbits. This result suggested an increase of AchE activity in the heart of H rabbits. In fact, in about 50–60% of vagal hyperreactive rabbits, overexpression of muscarinic receptors was associated with an increase in expression of the AchE gene in the heart and in enzyme activity in erythrocytes. This increase of AchE expression and activity appears at first glance paradoxical. Looking at individual data, while no overlap between B_max_ values obtained from H rabbits compared with controls was observed, there was some overlap for AchE expression and activity. Indeed, two groups of H rabbits were identified: a first one which displayed enhanced AchE levels, and a second one which did not. Taken together, these results indicate that the primary cardiac abnormality is in fact the overexpression of muscarinic receptors whereas AchE upregulation appears as a possible compensatory consequence of the latter.

Data of the maturation studies are in agreement with this assumption. Thus, muscarinic receptor expression remains stable between 5 and 7 weeks of age in hyperreactive rabbits while it is largely downregulated in normal rabbits. The concomittent increase in AchE level in hyperreactive animals may be a compensatory process opposing the functional consequences of the high muscarinic receptor expression to maintain the M_2_/AchE ratio. These alterations in H rabbits occurred at a very specific age (between the 5th and the 7th week after birth), at which abnormally high mortality was also observed. Therefore, stability of the M_2_/AchE ratio appears as the key determinant of survival at least at the age of 7 weeks. Further studies will establish whether the M_2_/AchE ratio is actually modified in deceased offsprings of H rabbits. If this were also true in SIDS, muscarinic receptor overexpression would be the link between vagal hyperreactivity and SIDS. We would then have come full circle.

Of note, similar patterns of changes were found whether M_2_ receptor expression and AchE levels were assessed in cardiac tissue or in blood cells, in which muscarinic receptors and AchE are known to be expressed [25]–[27]. Thus, it appears that cardiac abnormalities can be inferred with high confidence from M_2_ muscarinic receptor and AchE expression measurements in easily accessible blood cells, which may be of great practical interest. At least in our animal model, the muscarinic receptor expression level in peripheral mononuclear white blood cells appears as a reliable and easily measurable marker of vagal hyperreactivity and baroreflex dysfunctions.

Further investigations are needed to determine whether muscarinic receptors are also overexpressed in peripheral mononuclear white blood cells from patients with vagal syncopes and from SIDS – as observed in rabbits. If so, muscarinic receptor expression level in peripheral mononuclear white blood cells could become a reliable and easily measurable marker of risk among subjects exhibiting vagal or vasovagal syncopes, which could be of great clinical interest.

We previously demonstrated that inheritance of the vagal disorder in our rabbit experimental model is polygenic with a partial sex-limited character [12]. We therefore sought for a mutation on the muscarinic M_2_ receptor gene and indeed identified a single nucleotide mutation in the coding fragment of the M_2_ muscarinic receptor gene of 83% of the vagal hyperreactive rabbits. However, the mutation from a CCT into a CCG codon does not change the amino-acid sequence since both codons encode for the same amino-acid, *i.e.*, proline, and may then induce a quantitative rather than qualitative alteration in the M_2_ gene. Such a T→G mutation could create an exonic splicing enhancer site (ESE), interacting with the SF2/ASF splicing factor, and could simultaneously delete another ESE interacting with the SC35 splicing factor [28], [29]. ESE sequences are known to facilitate splicing through their interactions with various proteins [30]. The appearance of a new splicing site could lead to a qualitatively different gene product or, maybe more relevant in our model, to the overexpression of normal transcripts [31]. In agreement with the latter assumption, it is remarkable that the density in muscarinic receptors is much higher in rats, in which the reference codon sequence is CCG, compared to human and rabbits, in which the reference codon sequence is CCT [32].

In conclusion, we showed that overexpression of cardiac muscarinic receptors may play a critical role in the development of vagal hyperreactivity. The average AchE activity and expression were also increased in hyperreactive rabbits compared to controls, which could represent an attempt to oppose the increased muscarinic receptor density in order to maintain the sympatho-vagal balance. A same pattern of changes was detected in peripheral mononuclear white blood cells. Thus, in our animal model, muscarinic abnormalities in cardiac tissues could be inferred with high confidence from those measured in lymphocytes. Finally, vago-cardiac abnormalities detected in tissues from hyperreactive animals were similar to those detected in the hearts of SIDS. Altogether, these data raise the possibility that muscarinic receptor expression level in peripheral mononuclear white blood cells could become a reliable and easily measurable marker of risk of vasovagal syncopes and sudden death.
