# Correction: Constitutive Overexpression of Muscarinic Receptors Leads to Vagal Hyperreactivity

**DOI:** 10.1371/annotation/2747fb4d-de47-4e66-81f9-76b5f5ac4b90

**Published:** 2011-01-11

**Authors:** Angelo Livolsi, Nathalie Niederhoffer, Nassim Dali-Youcef, Walid Mokni, Catherine Olexa-Zorn, Jean-Pierre Gies, Luc Marcellin, Josiane Feldman, Pascal Bousquet

The affiliation for the 9th author was incorrect. The correct affiliations for Pascal Bousquet are: 1. Laboratoire de Neurobiologie et Pharmacologie Cardiovasculaire (EA 4438), Université de Strasbourg, Strasbourg, France; and 6. Centre d’Investigation Clinique, Hôpitaux Universitaires-INSERM, Strasbourg, France.

